# Enhanced susceptibility of cyclin kinase inhibitor p21 knockout mice to high fat diet induced atherosclerosis

**DOI:** 10.1186/1423-0127-16-66

**Published:** 2009-07-15

**Authors:** Ashwani K Khanna

**Affiliations:** 1 Department of Medicine (Cardiology), University of Maryland, Baltimore, USA

## Abstract

Cyclin kinase inhibitor p21 is one of the most potent inhibitors of aortic smooth muscle cell proliferation, a key mediator of atherosclerosis. This study tests if p2l deficiency will result in severe atherosclerosis in a mouse model. p21^-/- ^and strain matched wild type mice were fed with high fat diet for 21 weeks. Analysis for biochemical parameters (cholesterol, triglycerides) in serum and mRNA expression of CD36, HO-1, TGF-β, IFN-γ, TNF-α, PPAR-γ and NADPH oxidase components (p22^phox^, NOX-1 and Rac-1) was performed in aortic tissues by Real Time PCR. p21^-/- ^mice gained significantly (p < 0.01) more weight than wild type mice, triglycerides (p < 0.05) and cholesterol levels (p < 0.01) were more pronounced in the sera of p21^-/- ^compared to wild type mice fed with high fat diet. High fat diet resulted in significantly decreased TGF-β (p < 0.02), HO-l (p < 0.02) and increased CD36 (p < 0.03) mRNA expression in aortic tissues of p21^-/- ^mice compared to animal fed with regular diet. IFN-γ mRNA expression (235 ± 11 folds) increased significantly in high fat diet fed p21^-/- ^mice and a multifold modulation of PPAR-γ(136 ± 7), p22^phox^, NOX-1 and Rac-1 (15–35-folds) mRNA in aortic tissues from p21^-/- ^mice compared to the wild type mice. Severity of atherosclerotic lesions was significantly higher in p21^-/- ^compared to wild type mice. The results demonstrate that the deficiency of p21 leads to altered expression of pro-atherogenic genes, and severe atherosclerosis in mice fed with high fat diet. This opens the possibility of p21 protein as a therapeutic tool to control progression of atherosclerosis.

## Introduction

Atherosclerosis is one of the major causes of death in the modern world. Despite large efforts, its pathogenesis remains largely unclear. More recently, atherosclerosis is considered to be a disease of inflammation [1-4]. The precise events leading to inflammation/immune activation in atherosclerosis are not fully understood. Aberrant proliferation of smooth muscle cells is one of the key factors in the pathogenesis of atherosclerosis. Therefore, molecules that control cellular proliferation play a significant role in the understanding of underlying mechanisms of the pathogenesis of atherosclerosis. Transforming growth factor-beta (TGF-β) is one of the most potent inhibitor of smooth muscle cell proliferation [5], its expression decreases in atherosclerosis [6,7], highlighting its role in the pathogenesis of atherosclerosis [8]. Besides, these reported protective effects of TGF-β, its role in atherosclerosis is controversial because studies have demonstrated a causative role of TGF-β in the pathogenesis of atherosclerosis is reported based on the studies, which demonstrate that active TGF-β in smooth muscle cells promote lipid accumulation via increased synthesis [9-11] and deposition of proteoglycan in intima, known to be associated with increased atherosclerosis [12,13]. In another study, TGF-β expression was considered to be a determinant for the extent to which developing atherosclerotic lesions are stabilized by a collagen-rich fibrous cap and higher levels of TGF-β in SMC of stable lesions compared to the unstable lesions were observed [14]. These studies suggest that the role of TGF-β may be detrimental in atherosclerosis. Studies have shown that TGF-β induces expression of cyclin kinase inhibitor p21, which mediates its inhibitory effects [15]. Overexpression of p21 has been shown to reverse atherosclerosis in experimental models of atherosclerosis. [16]. In addition to inducing expression of protective molecules like p21, TGF-β also inhibits the expression of CD36, a scavenger of LDL [17]. We have earlier demonstrated 5 that the aberrant proliferation of smooth muscles due to the deletion of TGF-β gene was restored by p21. Therefore, it suggests that p21 play a significant role in the control of aberrant proliferation of smooth muscle cells, an essential event in the pathogenesis of atherosclerosis. Furthermore, the protective role of heme oxygenase-1 (HO-1) in atherosclerosis is also attributed to its induction of p21 [18]. Similarly, other studies have demonstrated that the positive effects of HO-1 are associated with increased expression of p21 that promotes the inhibition of smooth muscle cell proliferation and provides protection against atherosclerosis [19-21]. Also, atherosclerosis is considered to be due to a state of heightened oxidative stress [22] and p21 provides protection from oxidative stress [23,24]. Therefore, these studies were performed to understand the role of p21 on the pathogenesis of atherosclerosis using p21^-/- ^mice. The animals were fed either a high fat or regular diet. Aortic expression of TGF-β, CD36, HO-1, IFN-γ, PPAR-γ and NADOH oxidase components (p22^phox^, NOX-1 and Rac-1) was studied and histopathology was quantified. The results demonstrate that the modulation of p21 can assist in dissecting the events leading to the initiation and development of atherosclerosis.

## Materials and methods

### Mice and treatment

Mice (6 weeks old) lacking p21 (p21^-/-^) were bred from a breeding pair generously provided by Dr. Philip Leder, Howard Hughes Medical Institute, Boston. Strain matched (FVB) wild type mice were purchased from Taconic Labs. Wild-type (6 weeks old) and p21^-/- ^mice (24 each) were fed initially with regular diet, which consisted of <0.05% cholesterol, approximately 5% of animal fat without casein or sodium cholate, and then were fed with a standard atherogenic diet. The high-cholesterol diet from Dyets Inc. Bethlehem, PA USA) were used. The high cholesterol diet was continued for 21 weeks when a peak of atherosclerosis is achieved. All studies were performed with approved institutional IACUC protocols.

### Lipid profile

Sera was separated and stored at -80°C until analysis. Using specific kits from Wako Chemicals Richmond, VA, USA, levels of total cholesterol and triglycerides were quantified from sera samples obtained at study endpoint (21 weeks of high cholesterol diet)..

### Analysis of mRNA expression of pro-and anti-atherogenic intermediates in atherosclerosis

We performed real-time quantitative RT-PCR for IFN-γ, PPAR-γ and NADPH oxidase components (p22phox, NOX-1 and Rac-1) mRNA using a Bio-Rad iCycler system (Bio-Rad, Hercules, CA). RNAs were isolated from cardiac l tissues using a kit from Promega (Madison, USA) and reverse-transcribed into cDNAs by using a cDNA synthesis kit from invitrogen (Carlsbad CA). The amplification of specific mRNA expression was achieved by polymerase chain reaction (PCR) using specific primer sequences for **TGF-β; **sense: 5'-GGGACTA TCCACCTGCAAGA-3'; antisense: 5'-CACGTGCTGCTCCACTTTTA-3'; **CD36**; sense: 5'-AGATGCA GCCTCATTTCCAC-3'; antisense: 5'-GCCTTGGATGGAAGAACAAA-3'; **HO-1**; sense: 5'-TCCGATGGGTCCTTACACTC-3'; antisense: 5'-ATTGCCTGGATGTGCTTT TC-3' and **β-actin**; sense: 5'-TGACGGGGTCACCC ACACTGTGAACATCTA-3'; antisense, 5'-CTTGAAGCATTTGCGGTGGACGATGGAGGG-3'; **IFN-γ **sense 5'-TCTGGAGGAACTG GCAAAAG-3', antisense 5'-TTCAAGACTTCAAAGAGTC TGAGG-3'; **p22**^phox ^sense 5'-G CCATTGCCAGTGTGATCTA-3'; antisense: 5'-AATGGGAGTCCACTGCTCAC-3'; **NOX-1**; sense: 5'-GGCATCCCTTTACTCTG ACCT-3'; antisense: 5'-TGCTGCTCGAATATGAA TGG-3'; **Rac-1**; sense: 5'-GTA CATCCCCACCGTCTTTG-3'; antisense: 5'-CCCAGATTC ACTGGTTTTC-3' and **β-actin **sense: 5'-CCCAGCACAATGA AGATCAA-3' and antisense 5'-CG ATCCACACGGAGTACTTG-3. The primers were tested by running a regular PCR for 40 cycles at 95°C for 20 s and 60°C for 1 minute, and followed separating in ethidium bromide containing agarose gels. The real-time PCR was performed using a SYBR supermix kit (Bio-RAD), and running for 40 cycles at 95°C for 20 s and 60°C for 1 minute. The PCR efficiency was also examined by serially diluting the template cDNA and the melting curve data was collected to check the PCR specificity and proper negative controls were included in each assay. The mRNA level for each gene for each sample was normalized to β-actin mRNA and quantified using a formula; 2 [(Ct/β-actin – Ct/gene of interest)]. The results are expressed as fold difference in high fat diet fed compared to regular diet fed p21^-/- ^mice.

### Histology and Morphometry

The differences among the high cholesterol fed wild-type mice and p21^-/- ^mice regarding atherogenesis was evaluated quantitatively by microscopic examination the atherosclerotic lesions. Serial cross-sections at least in triplicate were cut through the aortic coronary artery region, beginning with the appearance of all three-valve cusps as described by Daugherty and Whitman [25]. The sections were stained with Hematoxylin-and-eosin and Oil Red O, and counter-stained with hemalum. The quantification of the atherosclerotic lesions was made by using an Olympus microscope and by computer-aided morphometry software Image J. The average lesion size for each mouse were calculated and converted in to percent of total area counted. Statistical analysis was performed by the use of statistical software (GraphPad, San Diego).

### Data analysis

Differences between groups were determined using two-tailed unpaired T test with significance considered present at a p value of less than 0.05. Statistical analysis was performed using a software program from GraphPad Software, Inc., San Diego, CA 92121 USA. The results are expressed as Mean ± SEM.

## Results

### Weight gain

**Figure 1:**
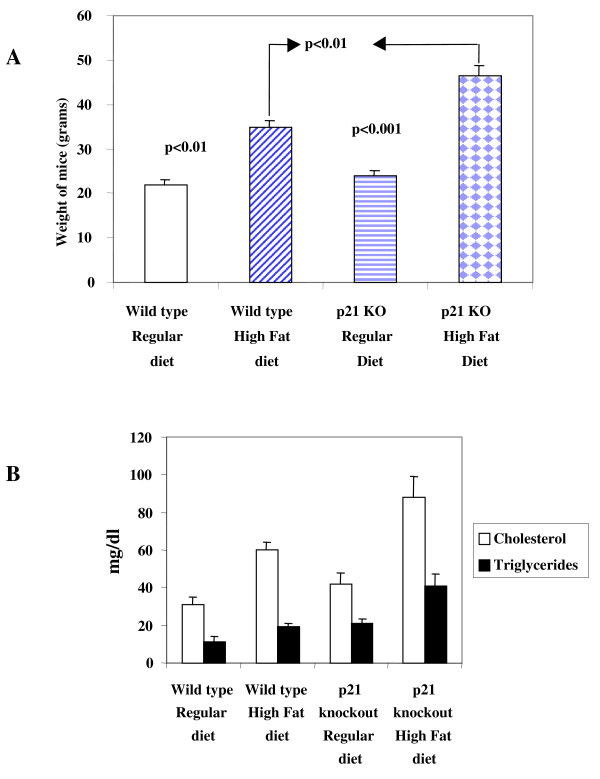
**Body weight (BW), plasma cholesterol, and plasma triglyceride levels in p21^-/- ^and wild type control mice**. **A**: Differential effect of high fat diet on weight gain in wild type mice (n = 8) and p21^-/- ^(n = 8) mice is shown. Values are means ± SEM, Weight gain in p21^-/- ^mice fed with high fat diet is compared to p21^-/- ^mice fed with regular diet and wild type mice fed with high fat diet. **B**: Differential effect on the effect of high fat diet on plasma levels of cholesterol (open bars) and triglycerides (closed bars) in p21^-/- ^and wild type mice fed with high fat and regular diet is shown.

Wild-type mice gained weight (22 ± 1.2 g vs 35 ± 1.5 g, p < 0.01) when fed with high fat diet, however weight gain in and p21^-/- ^was significantly higher (24 ± 1 vs 46.4 ± 2.3 g, p < 0.001) when fed with high fat diet. Interestingly, weight gain in p21^-/- ^mice was significantly higher (p < 0.01) compared to wild-type mice also fed with high fat diet (Figure 1A).

### Triglycerides and Cholesterol Levels

These results demonstrate that p21^-/- ^mice compared to their wild type counterparts were more prone to the effects of high fat diet. Circulating levels of cholesterol and triglycerides levels in sera of mice from each group were quantified (Figure 1B). Triglycerides levels increased in wild-type mice fed with high fat diet compared to mice fed with regular diet (11-± 3 vs 21 ± 2 mg/dl). However, the increase of triglycerides levels was more pronounced in sera of p21^-/- ^mice fed with high fat diet compared to regular diet (19 ± 2.5 vs 41 ± 6 mg/dl p < 0.001). A significant (p < 0.05) increase in triglycerides levels was observed when we compared levels of wild-type and p21^-/- ^mice. Similar results were obtained with quantification of cholesterol levels. The circulating levels of cholesterol increased in wild-type mice fed with high fat diet compared to mice fed with regular diet (31 ± 4 vs 60 ± 4 mg/dl p < 0.05). However, the increase of cholesterol levels was more pronounced in sera of p21^-/- ^mice fed with high fat diet compared to regular diet (42 ± 6 vs 88 ± 11 mg/dl p < 0.001). A significant (p < 0.05) increase in circulating levels of cholesterol was observed when levels in sera of wild-type and p21^-/- ^mice fed with high fat diet were compared.

### Effect of high fat diet on mRNA expression of anti-atherogenic (TGF-β, HO-1) and pro-atherogenic (CD36) in p21^-/- ^mice

#### TGF-β mRNA expression

**Figure 2:**
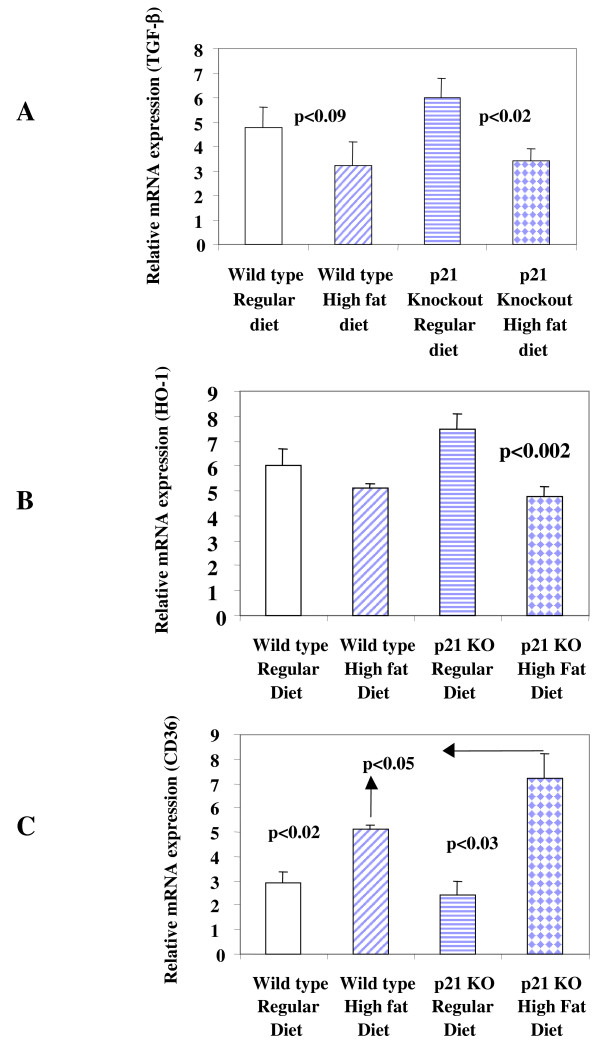
**Atherogenesis related gene expression in p21^-/- ^and wild type control mice**. Intra-aortic analysis of mRNA expression for anti-atherogenic TGF-β (**A**), HO-1 (**B**) and pro-atherogenic gene CD36 (**C**) is shown. Values presented are means ± SEM in wild type (n = 8) and p21^-/- ^mice (n = 8) fed with regular and high fat diet.

The expression of TGF-β mRNA in aortic tissues of mice fed with high fat diet was compared in mice fed with high fat and regular diet. Total RNA from aortic tissues was reverse transcribed to cDNA and amplified for TGF-β mRNA by PCR. In wild-type and p21^-/- ^mice, TGF-β mRNA expression decreased though in wild-type mice the decrease was not significant (Figure 2A). In p21^-/- ^mice a highly significant (p < 0.02) decrease was observed. We also have data on TGF-β mRNA expression in aortic tissues from p21^-/- ^mice fed with regular or high fat diet. A highly significant (p < 0.01) decrease in TGF-β mRNA was observed in high fat diet fed mice compared to mice fed with regular diet [Relative TGF-β mRNA expression (M ± SEM) n = 4, .5 ± 0.4 vs 3.6 ± 0.3).

#### CD36 mRNA expression

Based on its role in the pathogenesis of atherosclerosis, it was hypothesized that the expression of CD36 mRNA in aortic tissues of mice fed with high fat diet will be more than mice fed with regular diet. In wild-type and p21^-/- ^mice, CD36 mRNA expression increased but in p21^-/- ^mice a highly significant (p < 0.03) increase was observed (Figure 2B) compared to wild type mice. The expression of CD36 mRNA was significantly more (p < 0.05) in p21^-/- ^mice fed with high fat diet compared to wild-type mice also fed with high fat diet.

#### HO-1 mRNA expression

HO-1 has been shown to be protective in atherosclerosis, therefore, mRNA expression was studied in p21^-/- ^mice fed with high fat diet and compared to wild-type mice fed with similar diet. Total RNA from aortic tissues was reverse transcribed to cDNA and amplified for HO-1 mRNA by PCR. In both wild-type and p21^-/- ^mice HO-1 mRNA expression decreased but in p21^-/- ^mice a highly significant (p < 0.002) decrease was observed (Figure 2C).

### Effect of high fat diet on mRNA expression of IFN-γ, PPAR-γ and NADPH oxidase components (p22^phox^, NOX-1 and Rac-1) in p21^-/- ^mice

**Figure 3:**
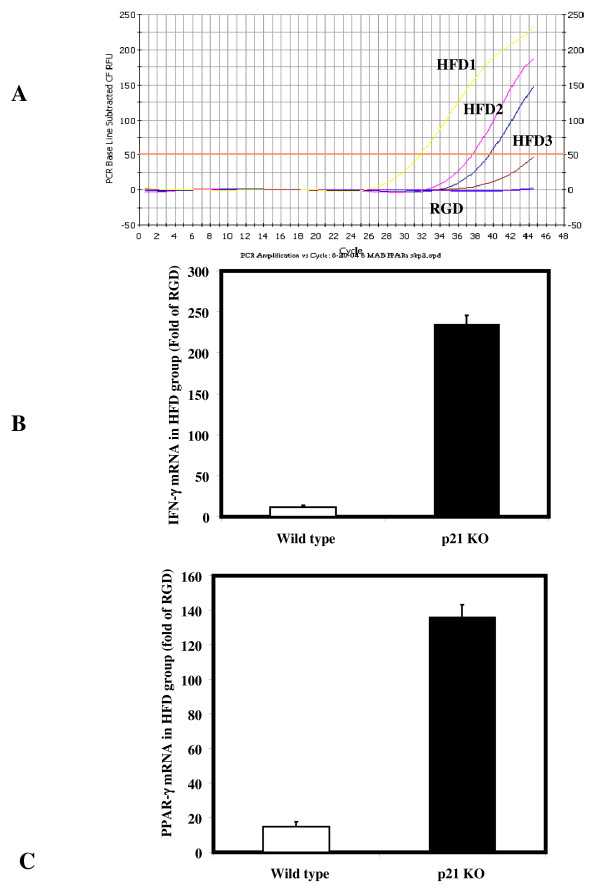
**Quantitative mRNA expression of IFN-γ, PPAR-γ and NADPH oxidase components (p22^phox^, NOX-1 and Rac-1) in aortic tissues from p21^-/- ^mice**. Amplification curves for IFN-g in RNA isolated from aorta from mice fed with high fat diet (HFD) and regular diet (RGD) are shown. Significantly higher Relative fold expression of IFN-γ (**B**), PPAR-γ (**B**) mRNA is shown in Wild type (open bars) and p21^-/- ^mice (closed bars). The values are means ± SEM of fold increase in high fat diet compared to the results obtained from mice fed with regular diet.

IFN-γ mRNA expression was studied by Real time PCR analysis. IFN-γ mRNA increased in p21^-/- ^mice fed with high fat diet compared to those fed with regular diet. These results amplification peaks in a real time PCR reaction (Figure 3A) show that IFN-γ mRNA was detectable at a very low levels in regular diet fed group compared to significantly higher (236 ± 11 fold) in p21^-/- ^mice compared to (12 ± 1.6 fold) in wild type mice fed with high fat diet (Figure 3B). To understand the role of peroxisome proliferator-activated receptors (PPARs) in the development of atherosclerosis, the expression patterns of mRNA of PPAR-gamma in aortic tissues from mice was investigated. Higher PPAR-γ mRNA was detected in aortas from p21^-/- ^mice (136 ± 7.3-folds) compared to wild type mice (15 ± 2.7) fed with either high fat diet (Figure 3C). PPAR-γ is likely to be an important regulator of monocyte/macrophage function with relevance for human atherosclerotic disease.

**Figure 4:**
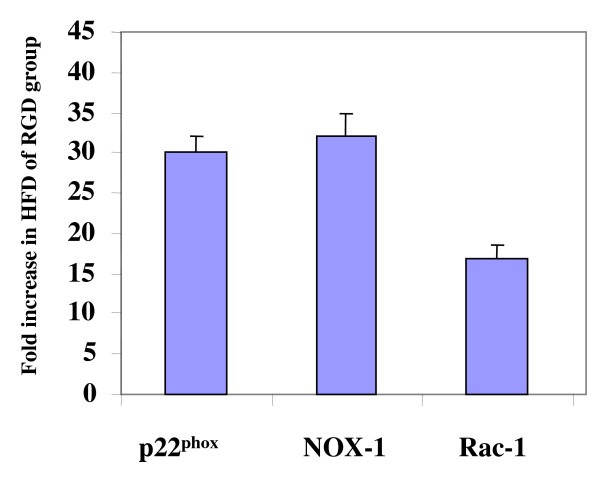
**Quantitative mRNA expression of NADPH oxidase components (p22^phox^, NOX-1 and Rac-1) in aortic tissues from p21^-/- ^mice**. The results of mRNA expression of NADPH oxidase components; p22^phox^, NOX-1 and Rac-1 are shown as fold increase in high fat diet (HFD) treated p21^-/- ^mice (n = 8) using results from regular diet (n = 8) treated mice as controls (RGD).

The results of mRNA expression of NADPH oxidase components is shown in Figure 4, the expression of p22^phox^, NOX-1 and Rac-1 increased 29 ± 1.6, 32 ± 2.1 and 15.6 ± 1.2 folds, respectively in high fat diet fed compared to regular diet fed p21^-/- ^mice. Significantly lower values were observed in wild type mice (data not shown).

### Histopathological Analysis

**Figure 5:**
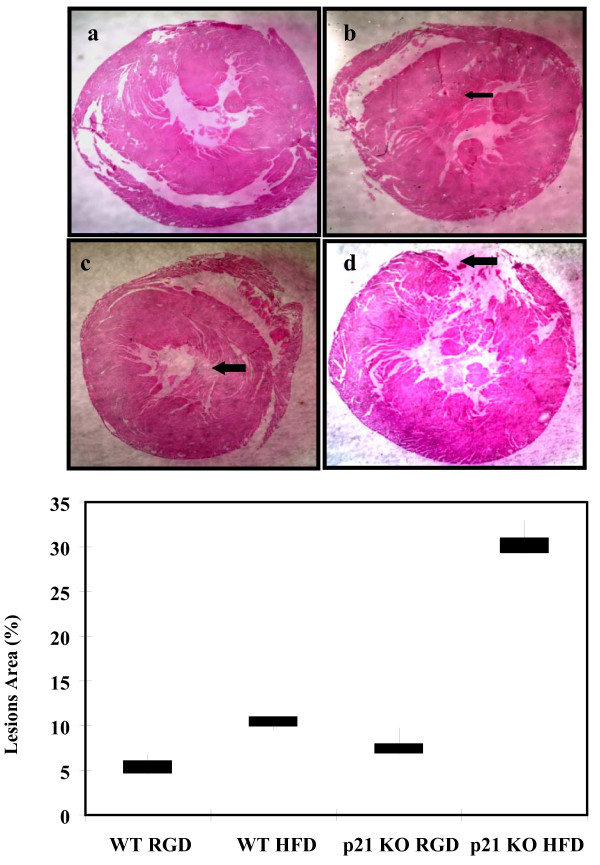
**Histology and morophometry of atherosclerotic lesions**. Photomicrographs of light microscopy of representative sections of the coronary artery tissues from p21^-/- ^and wild type mice. Magnification is ×20 for all tissues. Haematoxylin and eosin staining of in the coronary artery issues of wild type mice fed with regular diet (**a**), high fat diet (**b**); p21^-/- ^mice fed with regular diet (**c**) and high fat diet (**d**) is shown. **Black arrows **indicate areas with scar. The graph (**B**) shows the mean lesion area (values are Mean ± SEM) per mouse. *p < 0.01, high fat diet (HFD) vs regular diet (RGD), wild type mice and **p < 0.0001, high fat diet vs regular diet in p21^-/- ^mice.

The histological analysis of cross-sections cut through the aortic coronary artery region are shown Figure 5A. The results shows marked differences in p21^-/- ^mice fed with high fat diet (**d**) either compared to p21^-/- ^mice fed with regular diet (**c**) or wild type mice fed with high fat diet (**a**), a low power view of a coronary artery showing narrowing of the lumen by atheromatous plaque and scar areas (**black arrows**) are shown. The score of lesions are expressed as the percent lesions, quantified in aortas from wild type and p21^-/- ^mice fed with either regular diet or high fat diet and expressed as mean ± SEM. Percent area with aortic lesions was higher (4 ± 0.4% vs 10.2 ± 0.3%, p < 0.01) in wild type fed with high fat diet compared to regular diet. However, the extent of severity of atherosclerotic lesions was significantly more (8.4 ± 0.6% vs 30.9 ± 0.8%, p < 0.0001) in p21^-/- ^mice (Figure 5B).

## Discussion

The results from this study demonstrate that p21^-/- ^mice exhibited tendency towards increased atherosclerosis when fed with high fat diet. This included significantly more gain in weight than mice fed with regular diet. Similarly, p21^-/- ^mice fed with high fact diet showed significantly increased circulating levels of cholesterol and triglyceride compared to p21^-/- ^mice fed with regular diet and wild type mice fed with high fat diet. The results from gene expression analysis of pro- and anti-atherosclerotic molecules such as TGF-β, HO-1 and CD36 suggests that high fat diet differentially modulate these genes in p21^-/- ^and wild type mice in favor of atherogenesis. The aortic tissue mRNA of TGF-β decreased minimally in wild type mice fed with high fat diet but the decrease was significantly more in p21^-/- ^mice fed with high fat diet compared to same mice when fed with regular diet. In sharp contrast, intra aortic CD36 mRNA was significantly higher in p21^-/- ^mice fed with high fat diet compared to same mice fed with regular diet and wild type mice fed with high fat diet. These studies suggest such a sequential relationship between TGF-β and CD36 in atherosclerosis. CD36 was recognized more than two decades ago as a membrane glycoprotein, initially considered to be a receptor for thrombospondin-1 (TSP-1). However it is now realized to be a multi ligand scavenger because besides TSP-1, its ligands are long-chain fatty acids, modified LDL, retinal photoreceptor outer segments, malarial parasite, malaria-parasitized erythrocytes, sickle erythrocytes, anionic phospholipids, apoptotic cells, and collagens I and IV [26,27]. Among the various activities of CD36 are cell attachment, motility, proliferation, and regulation of protease activity, angiogenesis and above all TGF-β activation. Therefore, CD36 is also termed as multi-ligand scavenger receptor and one of the important pathologic functions of scavenger receptors, related to foam cell formation and the pathogenesis of atherosclerosis, is recognition and internalization of oxidatively modified LDL. Furthermore, the most compelling data supporting the role for CD36 in foam cell formation and atherosclerosis are from studies with CD36-knockout mice. Macrophages isolated from these animals were found to be defective in uptake of oxLDL and foam cell formation. Also, breeding the CD36 deficient mice with proatherogenic ApoE^-/- ^mice resulted in significant protection of animals from lesion development. Animals fed a Western diet showed a >70% reduction in aortic lesion size and distribution [28]. TGF-β has been shown to inhibit expression of CD36 [21,29]. A deficiency in TGF-β, which also results in deficiency of p21 5 could result in increased expression of CD36 leading to severe atherosclerosis. The results from these experiments demonstrate such a reciprocal profile of CD36 and TGF-β mRNA, suggesting that high fat diet in p21^-/- ^mice tilted the balance towards atherogenesis.

The analysis of HO-1 mRNA indicate that similar to TGF-β mRNA expression, its expression decreased significantly in p21^-/- ^mice fed with high fat diet compared to regular diet. The decrease in wild type mice also fed with high fat diet was minimal, suggesting the direct effect of p21 deficiency on HO-1 mRNA expression in aortic tissues of mice fed with high fat diet. HO-1 is an inducible form of heme oxygenase and possesses a variety of adaptive responses against oxidative stress. A number of factors, which include ischemia/reperfusion, hypertension, proinflammatory cytokines, or oxidized LDL, induce expression of HO-1 [30]. The HO reaction involves degradation of heme leading to its conversion to into biliverdin, carbon monoxide, and free iron [31,32]. These events are considered to provide protective roles against stresses in the vascular wall caused by a variety of pathological changes. Oxidative alteration of lipoproteins in vascular wall, which supposedly leads to the initiation and development of atherosclerosis mediate the relationship of HO-1 with atherosclerosis. HO-1 modulation in rabbits [33] and mice [34,35] demonstrated anti-atherogenic effects of HO-1.

Our findings that the aortic mRNA expression of HO-1 in p21^-/- ^mice decreases when fed with high fat diet and develop atherosclerosis are of significance, since with reference to atherosclerosis there are functional similarities between p21 and HO-1. Both these molecules promote cell arrest, one of the key step in preventing smooth muscle cell proliferation and atherosclerosis. Furthermore, p21 seems to be of more significance, since growth inhibition and cell-cycle arrest in HO-1 expressing cells was shown to be associated with induction of p21 [21]. Interestingly, HO-1 deficient cells lack or have reduced p21 expression, in the present study, opposite seems to be true, in p21^-/- ^mice, HO-1 expression significantly decreased when fed with high fat diet.

Atherosclerosis is a disease of inflammation. [1-4], in this study; we demonstrate that p21^-/- ^mice when fed with high fat diet developed significantly severe atherosclerosis compared to same mice fed with regular diet or wild type mice fed with high fat diet. To understand if inflammation was responsible in these mice, IFN-γ mRNA was analyzed in aortic tissues. The results demonstrate that high fat diet in p21^-/- ^mice resulted in significantly increased IFN-γ mRNA compared to the same mice fed with regular diet or wild type mice fed with high fat diet. These results suggest that p21 deficiency results in an increased inflammatory response to a high fat diet. Exogenous IFN-γ has been shown to enhance high fat diet induced atherogenesis in ApoE^-/- ^mice [36,37] and IFN-γ and TNF-α knockout mice did not develop severe atherosclerosis when fed with a diet with high cholesterol [38,39]. There is a large body of evidence suggesting a potent role of inflammation in atherosclerosis. T lymphocytes isolated from atherosclerotic plaques were found to have increased expression of IFN-γ and HLA class II molecules indicating the state of T cell activation [40-42]. Besides IFN-γ, PPAR-γ mRNA in the aortic tissues of p21^-/- ^mice increased significantly in response to high fat diet compared to regular diet. Though the precise role of PPAR-γ in atherosclerosis is not clear, it has been linked to the development of inflammation and studies [43] have shown its increased expression in macrophage foam cells of human atherosclerotic lesions and endothelial cells of human carotid arteries.

Oxidative stress has been shown to be a key factor in the pathogenesis of atherosclerosis. However, there is a lack of sufficient knowledge delineating the precise molecular events and the mediators involved in this process. The gene expression for NADPH oxidase components (p22^phox^, NOX-1 and Rac-1) was studied in aortic tissues from p21^-/- ^mice fed with high fat diet and compared with same mice fed with regular diet. It is conceivable that the increased activity of NADPH oxidase components reflected by multifold increased mRNA expression contributed to severe atherosclerosis in these mice. Increased expression of p22^phox ^has been associated with increased vascular smooth muscle cell proliferation and increased expression of gp91^phox ^and p22^phox ^mRNA was associated with the severity of atherosclerosis [22]. The results from this study suggest that in the absence of p21, its protective effects on oxidative stress [23,24] are lost that result in increased NADPH oxidase activity i.e. oxidative stress and severe atherosclerosis in p21^-/- ^mice fed with high fat diet compared to the regular diet.

These studies accumulatively provide direct evidence for a potential protective role of p21 *in vivo *for vascular disorders involving proliferative disorder specifically atherosclerosis. This may be due to aberrant smooth muscle cell proliferation in these mice. p21 has been shown to inhibit both the migration and proliferation of smooth muscle cells [44] and adenovirus mediated p21 gene expression in rat vascular smooth muscle cells inhibited proliferation [45]. Also in a porcine balloon arterial model, adenovirus-mediated transfer of the p21 gene resulted in 35% reduction of *in vivo *cell proliferation and intimal thickening [46]. The inhibition of p27, another potent cyclin inhibitor, blocked Ang II induced hypertrophy and promoted hyperplasia indicating a role of cell cycle control in the pathogenesis of atherosclerosis and other vascular disorders [47]. These studies indicate a role of cell cycle control and most significantly of p21 in the arrest of cellular growth in atherosclerosis. Our own studies [48,49] have demonstrated that p21 overexpression reduces mitogen-induced lymphocyte proliferation and inflammation, one of the key mediators in the pathogenesis of atherosclerosis. A number of studies have demonstrated the role of p21 as a protective agent in atherosclerosis. Inhibition of Akt pathway resulted in decreased SMC proliferation and decrease in p21 expression abolished this inhibition [50]. These results demonstrated that p21 may be an integral part of the events leading to the inhibition of SMC proliferation. During progesterone-induced inhibition of rat aortic smooth muscle cell proliferation the expression of CDK2 and CDK4 and of p21 and p27 increased. NSAIDs aspirin, sodium salicylate, diclofenac, ibuprofen, indomethacin and sulindac induce a dose-dependent inhibition of proliferation in rat A10 VSMCs and the expression of p21 and p27 were increased [51] and tranilast-mediated inhibition of SMC was due to the increased expression of p21 [52]. Tranilast efficiently inhibited the smooth muscle cell proliferation but not those isolated from p21^-/- ^mice. The *in vivo *experiments also confirmed the role of p21 in limiting SMC proliferation, since the administration of tranilast significantly reduced the neointimal VSMC hyperplasia in wild-type mice but not in p21^-/- ^mice. IL-β induced SMC proliferation resulted in decreased p21 expression [53] Statins have been shown to have multiple activities in vascular disorders including atherosclerosis. The inhibition of the mitogen-induced proliferation of microvascular endothelial cell by Cerivastatin, an inhibitor of 3-hydroxy-3-methylglutaryl coenzyme A reductase, which inhibits the biosynthesis of cholesterol and its precursors: farnesyl pyrophosphate and geranylgeranyl pyrophosphate (GGPP) was associated with the increase of p21 expression. Most interestingly adenoviral mediated overexpression of p21 in hypercholesterolemic ApoE^-/- ^mice and resulted in a significant reduction of restenosis in these mice [54]. An inhibition of TSP-1 by a neutralizing A4.1 anti-TSP1 antibody prevented proliferation of serum-stimulated VSMCs. and was followed by a significant induction of p21 expression in A4.1-treated VSMCs [55]. Therefore, the results showed that p21 played an important role in TSP1-mediated control of cellular proliferation.

In summary these studies suggest that p21 is protective in atherosclerosis, though mechanism of its effect may not be completely clear. Based on our published studies on the anti-inflammatory effects of p21 [56] and the increased inflammation in p21^-/- ^mice, it can be speculated that p21 deficiency in the presence of high fat diet resulted in the development of atherosclerosis. In a number of other studies [57], efficacy of overexpression of p21 as an anti-inflammatory agent has been documented that includes experimental models of arthritis and systemic lupus erythmatosus (SLE). There is only one study with opposite effects than the data presented in this study. The authors [58] showed that p21 is proatherogenic molecule since inactivation of p21 protected against atherosclerosis by stimulating apoptosis, enhancing inflammation etc. However, there is no proven direct relationship between either facilitation of inflammation or phagocytic action of macrophages. Studies have strongly suggested that vascular proliferation and inflammation are linked [59] and coupled with the notion that cell cycle control and inflammation are also connected, makes extremely impossible to think that the deficiency of p21 could result in aggravated atherosclerosis. Initial studies have shown that an initial insult to endothelium results in release of growth factor and cytokines that stimulate the proliferation of smooth muscle cells. Interestingly, proliferation of smooth muscle cells remains one of the key events in the pathophysiology of atherosclerosis. The strongest evidence is provided by the studies demonstrating that the proliferation of SMC is limited as a consequence to the inhibition of cell cycle progression by modulation of p21 or antisense oligonucleotides (ODNs) against c-myc [5]. These studies clearly and directly demonstrate the protective but not the pro-atherogenic properties of p21.

Therefore, based on a large number of supportive studies suggesting multiple positive effects including hematopoiesis, carcinogenesis, nephrotoxicity and inflammation, it is highly unlikely that p21 deficiency will lead to aggravated atherosclerosis. In contrary, p21 deficiency, as observed in this study, will result in increased inflammation and more severe atherosclerosis. Besides, a number of studies, which have demonstrated that modulation of p21 alter cellular proliferation and inflammation, our recent studies have uniquely demonstrated that recombinant p21 protein localizes into nucleus of lymphocytes, inhibit cellular proliferation and inflammation [56]. Therefore, based on the anti-inflammatory and anti-proliferative effects of p21, we speculate a therapeutic role of p21 in limiting the development and progression of atherosclerosis.

## Competing interests

The author declares that they have no competing interests.
